# The diagnostic value of miR-340-5p in pediatric ulcerative colitis and its molecular mechanism by targeting *MAP3K2* to modulate intestinal epithelial cell dysfunction

**DOI:** 10.1186/s41065-025-00597-z

**Published:** 2025-11-19

**Authors:** Fanting Meng, Xianlong Han, Lingxia Ge, Nan Guan

**Affiliations:** 1 Department of Gastroenterology, Children’s Hospital of Nanjing Medical University, Nanjing City, Jiangsu Province 210000 China; 2 Department of Anorectal, Qingdao Hiser Hospital Affiliated of Qingdao University (Qingdao Traditional Chinese Medicine Hospital), Qingdao City, Shandong Province 266033 China; 3 Department of Obstetrics and Gynecology, Guangdong Provincial Key Laboratory of Major Obstetric Diseases, Guangdong Provincial Clinical Research Center for Obstetrics and Gynecology, Guangdong-Hong Kong-Macao Greater Bay Area Higher Education Joint Laboratory of Maternal-Fetal Medicine, Guangzhou City, Guangdong Province China; 4 Department of Pediatrics, Guangzhou Key Laboratory of Neonatal Intestinal Diseases, The Third Affiliated Hospital of Guangzhou Medical University, Guangzhou City, Guangdong Province China; 5 Department of Pediatrics, Affiliated Hospital of Gansu Medical College, No.296, Kongtong East Road, Pingliang City, Gansu Province China

**Keywords:** MiR-340-5p, Pediatric UC, MAP3K2, Inflammation, Oxidative stress

## Abstract

**Background:**

The incidence of pediatric ulcerative colitis (UC) is increasing yearly, and it is urgent to explore precise diagnostic biomarkers and molecular mechanisms. This study aims to investigate the diagnostic value of miR-340-5p in pediatric UC and its molecular mechanism mediated through targeting *MAP3K2*.

**Methods:**

Eighty-five pediatric UC patients and 50 healthy controls were enrolled. The expression levels of miR-340-5p and *MAP3K2* were detected by qRT-PCR, and Pearson’s correlation analysis was conducted. An in vitro model was established by inducing HT-29 cells with dextran sulfate sodium. The target was verified by dual-luciferase assay. Flow cytometry, ELISA, and oxidative stress detection were used to verify the cellular functions regulated by the miR-340-5p/*MAP3K2* axis.

**Results:**

In pediatric patients with UC, miR-340-5p was significantly downregulated and negatively correlated with PUCAI, CRP, and ESR (*P* < 0.0001). Additionally, the diagnostic area of miR-340-5p under the ROC curve was 0.908. Mechanistically, miR-340-5p directly interacts with *MAP3K2*, leading to suppressed expression of its mRNA and protein. Functional experiments revealed that miR-340-5p overexpression reversed DSS-induced exacerbated cell apoptosis, reduced levels of TNF-α, IL-6, IL-17, IL-1β, and MDA, and increased GSH. Conversely, the beneficial effects of miR-340-5p were attenuated by oe-*MAP3K2* overexpression.

**Conclusions:**

miR-340-5p may serve as a diagnostic biomarker for pediatric UC and exerts its effects by targeting *MAP3K2* to regulate cell apoptosis, inflammatory responses, and oxidative stress.

**Supplementary Information:**

The online version contains supplementary material available at 10.1186/s41065-025-00597-z.

## Background

Ulcerative colitis (UC), a chronic relapsing inflammatory disorder affecting the colonic mucosa, has witnessed a steady rise in both incidence and prevalence in recent years [25, 28]. In children, more than one-third of patients with ulcerative colitis often require hospitalization due to acute severe ulcerative colitis (ASUC), which is a higher proportion compared with adult patients. This high hospitalization rate is often related to difficulties in obtaining a clear diagnosis or experience diagnostic delays [3, 31]. The precise etiology of UC remains incompletely understood, with its pathogenesis thought to involve a complex interplay of various factors, such as infectious agents, immune system dysregulation, genetic susceptibility, and environmental influences [7, 10]. In addition, long-term UC may increase the risk of colorectal cancer (CRC), and targeted cancer cell therapy is a relatively novel treatment approach [19, 34]. Currently, for pediatric patients, the main therapeutic approaches focus on modulating the inflammatory response [8] and oxidative stress to control symptoms and prevent disease recurrence. Therefore, it is urgent to search for biomarkers for the accurate diagnosis of pediatric UC and to explore the underlying biological mechanisms of its development.

MicroRNAs (miRNAs) serve as pivotal regulators of multiple genes and signaling pathways engaged in the pathophysiology of inflammatory and autoimmune diseases [30]. Recent years have seen growing evidence that miRNAs participate in UC progression via inflammatory responses and other pathways. It is worth noting that studies on competitive endogenous RNA networks have predicted and validated through RT-PCR that significant dysregulation of miR-340-5p may play a key role in the pathogenesis of UC [13, 23, 42]. Furthermore, miR-340-5p exhibits anti-inflammatory and antioxidant effects in various contexts, including neuritis, diabetic cardiomyopathy, and acute pancreatitis [5, 33, 48]. Research has demonstrated that mitogen-activated protein kinase kinase 2 (*MAP3K2*) is essential for CD4 + T cell-mediated inflammation in the intestine, and Th1 cells regulate colitis severity through *MAP3K2* [39]. Notably, *MAP3K2* promotes the formation of an inflammatory microenvironment in intestinal stromal cells and is involved in regulating Th1 cell differentiation, making it a key molecule of significant research value in the pathogenesis of UC. Although many studies have identified immune-related gene features in active UC, the diagnostic value of miR-340-5p in pediatric UC patients and its molecular mechanism, particularly how it regulates *MAP3K2* to modulate inflammatory response, cell apoptosis, and oxidative stress, remain largely unclear.

In this study, miR-340-5p was selected as the research target to explore its clinical significance in the diagnosis of pediatric UC. By identifying its downstream target genes and elucidating the molecular regulatory mechanisms, this research aims to provide novel insights and therapeutic strategies for pediatric UC.

## Methods

### Study subjects

Between January 2021 and December 2023, this study consecutively recruited 85 pediatric UC patients who received treatment at the Affiliated Hospital of Gansu Medical College throughout this period. Additionally, 50 age- and gender-matched healthy children, who were enrolled during the same period at our hospital’s pediatric health examination center, served as the control group. The matching was performed on a group-level basis to ensure overall distributions of age and gender were comparable between the UC patient group and the control group. These control subjects were primarily children visiting the hospital for routine health check-ups. They were confirmed to have no history of gastrointestinal diseases, infections, or immunodeficiency disorders. Furthermore, all control subjects had normal results in laboratory tests including complete blood count, C-reactive protein, and erythrocyte sedimentation rate. This approach ensured that the controls and UC patients were drawn from the same geographical region and healthcare system, thereby minimizing potential confounding factors related to regional lifestyle or medical practice differences. Throughout the entire period, all clinical data and samples were collected from the subjects at the time of enrollment.

Exclusion criteria included: (1) coexisting infections (such as CMV, EBV) to prevent intestinal inflammation caused by other pathogens, (2) Crohn’s disease, infectious colitis, and celiac disease to reduce confusion caused by symptoms similar to pediatric UC, and (3) pediatric inflammatory bowel disease, immunodeficiency disease, and use of immunosuppressants in the past 4 weeks, as immunosuppressants were common treatment drugs for pediatric UC. Clinical indicators were collected from all subjects based on age and gender to analyze the association with pediatric UC. White blood cell count (WBC) and platelet count (PLT) reflected the systemic inflammatory status and immune activation degree of UC patients. Hemoglobin (Hb) was used to evaluate chronic intestinal bleeding or inflammatory suppression of hematopoiesis. Fecal calprotectin could effectively distinguish between organic and functional intestinal diseases and be used to evaluate mucosal healing status [17]. Disease activity was evaluated using the Pediatric Ulcerative Colitis Activity Index (PUCAI) [1]. All experimental procedures in this study were strictly conducted in accordance with relevant academic ethical and technical standards. The clinical sample-related protocols were approved by the Ethics Committee of the Affiliated Hospital of Gansu Medical College. Written informed consent was obtained from the guardians of all participating subjects, and the experimental design and implementation fully complied with the ethical principles for medical research involving human subjects as outlined in the Declaration of Helsinki. For in vitro cell experiments (including human colonic epithelial cell culture, transfection, and functional assays), all procedures followed the Standard Operating Procedures (SOP) for international cell biology research, ensuring legitimate cell sources, reproducible experimental processes, and compliance with biosafety requirements.

### Construction of DSS cell damage model

In this study, human colonic epithelial HT-29 cells (ATCC, USA, RRID: CVCL_0320) were utilized. The human colorectal adenocarcinoma cell line HT-29 was used in this study [26]. HT-29 is a widely utilized in vitro model for UC research, as it retains characteristics of intestinal epithelial cells and can be stimulated to produce various pro-inflammatory cytokines [16]. Its relevance lies in its ability to mimic the pathophysiological state of the intestinal epithelium in UC, particularly in studies focusing on epithelial barrier function, inflammatory responses, and the mechanisms of drug action [29]. The cells were cultured in DMEM medium (Gibco, USA, #10566016) containing 10% fetal bovine serum (FBS, Gibco, USA, #10270106) and 1% penicillin streptomycin (Gibco, USA, #15140122), at 37 °C and 5% CO_2_ until 15–25 passages. A UC-like injury model was established by treating cells with 2% dextran sulfate sodium (DSS, MP Biomedicals, #02160110-CF, Molecular Weight: 36,000–50,000) for 24 h. DSS is a sulfated polysaccharide that can reliably and repetitively induce pathological features similar to human UC [46]. It was widely used to construct UC in vitro models by directly disrupting the intestinal epithelial barrier, inducing inflammatory responses, and cell apoptosis [37, 45].

### Cell transfection

Cells were seeded into 6-well plates 24 h prior to transfection. Transfection was initiated when cell confluency reached 70%−80% using Lipofectamine 3000 (Invitrogen, USA; #L3000015) according to the manufacturer’s instructions. The transfection procedures involved 50 nM of miR-340-5p mimic (synthesized by Beijing Tsingke Biotechnology Co., Ltd.; miRBase: MIMAT0004692; sequence: 5′-UUAUAAAGCAAUGAGACUGAUU-3′) or a scrambled negative control (NC), together with 1 µg/well of MAP3K2 overexpression vector (oe-MAP3K2) or empty vector control (oe-NC). For rescue experiments, oe-MAP3K2 (1 µg/well) or oe-NC (1 µg/well) was co-transfected with the miR-340-5p mimic (50 nM). Transfection efficiency was evaluated 48 h post-transfection: qRT-PCR was used to detect miR-340-5p and MAP3K2 mRNA expression levels; for fluorescently labeled vectors, fluorescence intensity was quantified by fluorescence microscopy or flow cytometry. The oe-MAP3K2 plasmid was generated by cloning the full-length coding sequence (CDS) of human MAP3K2 (GenBank: NM_002406.5) into the pcDNA3.1(+) vector (Invitrogen, USA; #V79020). The inserted sequence was verified by Sanger sequencing (Beijing Tsingke Biotechnology Co., Ltd, China). The oe-NC control consisted of the empty pcDNA3.1(+) backbone (Invitrogen, USA; #V79020).

### Real-time fluorescent quantitative PCR

Total RNA was isolated from serum and cells using the miRNeasy Mini Kit (Qiagen, DE, #217004). For miRNA, cDNA synthesis was performed with the TaqMan MicroRNA Reverse Transcription Kit (Applied Biosystems, USA, #4366596), using U6 as the internal reference. For mRNA, cDNA was synthesized using the PrimeScript RT Kit (TaKaRa, Japan, #RR036A) with *GAPDH* as an internal control (Table S1). Use PrimeScript RT reagent kit (TaKaRa, Japan, #RR036A) on QuantStudio 5 system (Applied Biosystems, #A28567, USA) and perform 40 cycles of denaturation at 94 °C for 30 s, annealing at 65 °C for 30 s, and extension at 65 °C for 1 min, calculated relative expression levels using the 2^−ΔΔCt^ method. This study used 3 biological replicates and 3 technical replicates.

### Western blot

Extract total protein using RIPA lysis buffer containing 1% PMSF, incubate on ice for 30 min, centrifuge at 12,000×g for 15 min at 4 °C, and collect the supernatant. Quantify protein concentration using BCA assay kit (Thermo Fisher Scientific, USA, # 23225). Equivalent amounts of protein 20–40 µg were separated by 10% SDS-PAGE and then transferred onto PVDF membrane by electrophoresis. The membrane was blocked with 5% skim milk in TBST at room temperature for 1 h, then incubate overnight with primary antibody *MAP3K2* (Abcam, UK, # ab155187, RRID: AB_2893848, 1:1000) and GAPDH antibody (Abcam, UK, #ab9485, RRID: AB_307274, 1:5000) at 4 °C. After washing TBST three times, incubate at room temperature with HRP labeled secondary antibody (Abcam, UK, #ab2116, RRID: AB_302851, 1:5000) for 1 h. After TBST membrane washing, ECL reagent combined with chemiluminescence imaging system was developed; ImageJ quantitative band grayscale, the relative expression level of the target protein is represented by its grayscale ratio to the internal reference. This study used 3 biological replicates and 3 technical replicates.

### Dual luciferase reporter gene experiment

The wild-type (WT) and mutant (MUT) sequences of the *MAP3K2* 3’UTR containing the putative miR-340-5p binding site were cloned into the pmirGLO vector (Promega, USA, #E1330). HEK293T cells (Abcam, UK, #ab282205, RRID: CVCL_0063) were co-transfected with the constructed reporter vectors and miR-340-5p mimic/negative control (NC). Following a 48-hour incubation, luciferase activity was measured using the Dual-Luciferase Reporter Assay System (Promega, USA, #E1910). As one of the “gold standards” for miRNA target gene validation, luciferase could effectively reflect the direct targeted binding relationship between miR-340-5p and *MAP3K2* [2]. This study used 3 biological replicates and 3 technical replicates.

### Apoptosis analysis

HT-29 cells were collected 48 h after transfection, washed twice with pre cooled PBS, and resuspended at a cell concentration of 1 × 10^6^ cells/mL in 1×Binding Buffer using the Annexin V-FITC/PI (BD Biosciences, USA, #556547) kit. 5µL Annexin V-FITC and 5µL PI were added to each tube, and incubated at room temperature in the dark for 15 min. The apoptosis rate was immediately analyzed using a BD FACSCanto II flow cytometer (BD Biosciences, USA, #654367). During analysis, the FSC-A/SSC-A scatter plot was first used to gate and exclude debris, thereby defining the single-cell population. Subsequently, cell populations were distinguished based on fluorescence signals: Annexin V^−^/PI^−^ (viable cells), Annexin V^+^/PI^−^ (early apoptotic cells), Annexin V^+^/PI^+^ (late apoptotic cells), and Annexin V^−^/PI^+^ (necrotic cells). The total apoptosis rate was calculated as the sum of the percentages of early and late apoptotic cells. Three independent experiments were conducted for cell apoptosis analysis, with blank and positive controls set up for each experiment. This study used 3 biological replicates and 3 technical replicates.

### ELISA detection of inflammatory factors

The levels of TNF-α, IL-6, IL-17, and IL-1β in cell culture supernatants were determined using specific ELISA kits (Thermo, USA; #EH3TNFA, #EH2IL6, #EHIL17, #EHIL1B) following the manufacturer’s protocols. Samples and standards were assayed in duplicate. After the incubation and substrate reaction steps, absorbance was measured at 450 nm. Cytokine concentrations were calculated based on the respective standard curves.

### Caspase-3 activity

Caspase-3 activity (Abcam, UK, #ab39401) was measured using a colorimetric assay. In a 96-well plate, 50 µL of cell lysate was added to 50 µL of diluted substrate working solution and gently mixed, with blank and negative controls established. The absorbance was measured at 405 nm after incubation in the dark at 37 °C for 2 h. This study used 3 biological replicates and 3 technical replicates.

### Detection of oxidative stress indices

After cell collection, measure the MDA (#A003-1) content and GSH (#A006-2-1) antioxidant marker activity according to the manufacturer’s protocol provided by the kit (NJBCI, China). Follow the instructions of the SOD (#A001-1–2) and T-AOC (#A015-1–2) detection kit (NJBCI, China) for testing. Simultaneously test the blank control and positive control to ensure the accuracy of the test results. This study used 3 biological replicates and 3 technical replicates.

### Statistical method

Data analysis and graphing were performed using GraphPad Prism 9.0 (RRID: SCR_002798) and SPSS (RRID: SCR_002865). The normality of all continuous variables, including age, white blood cell count (WBC), platelet count (PLT), hemoglobin (Hb), C-reactive protein (CRP), fecal calprotectin, miR-340-5p expression level, and the Pediatric Ulcerative Colitis Activity Index (PUCAI) score, was assessed using the Shapiro-Wilk test. All these variables showed no significant deviation from normality (*P* > 0.05); therefore, they are presented as mean ± standard deviation. Homogeneity of variances was verified using Levene’s test. For comparisons between two groups, an independent samples t-test was used. For comparisons among multiple groups, one-way analysis of variance (ANOVA) was applied when the assumption of homogeneity of variance was met. If the ANOVA result was significant (*P* < 0.05), Tukey’s post-hoc test was used. For data that violated the assumption of homogeneity of variance, Welch’s ANOVA was employed, followed by the Games-Howell post-hoc test. Categorical variables (e.g., gender distribution) were compared using the chi-square test. The Pearson product-moment correlation coefficient was used to evaluate the linear relationships between serum miR-340-5p expression levels and clinical parameters (PUCAI score, CRP, and fecal calprotectin). A receiver operating characteristic (ROC) curve was constructed to assess the diagnostic potential of miR-340-5p in distinguishing UC patients from healthy controls. The area under the curve (AUC) and its 95% confidence interval (CI) were calculated. The Venn diagram was generated using Venny 2.1 (RRID: SCR_016561). All in vitro cell experiments were conducted with three independent biological replicates (*n* = 3), each including three technical replicates. The data presented for statistical analysis are based on the means of these biological replicates, while technical replicates were used to verify assay stability. A two-sided *P*-value < 0.05 was considered statistically significant.

## Results

### Clinical baseline information analysis

Clinical data analysis revealed that the mean ages of the HC and UC groups were 9.22 ± 2.11 and 8.99 ± 2.12, respectively. Among them, white blood cells, platelets, hemoglobin CRP, erythrocyte sedimentation rate, and fecal calprotectin showed extremely significant differences between healthy individuals and UC patients (*P* < 0.0001). The PUCAI score in the UC group was 30.66 ± 14.05. (Table 1).

**Table 1:** Basic clinical information

Factor	HC (*n* = 50)		UC (*n* = 85)	*P*	*Mean Differences 95% CI*
Age (year)		9.22 ± 2.11	8.99 ± 2.12	0.541	−0.23 (−0.98 to 0.52)
WBC (10 ^9^ /L)		6.94 ± 1.19	9.60 ± 2.38	< 0.0001	2.65 (1.93 to 3.36)
PLT (10 ^9^ /L)		287.90 ± 46.57	321.10 ± 45.65	< 0.0001	33.19 (16.98 to 49.41)
Hb (g/L)		132.10 ± 8.51	111.70 ± 9.38	< 0.0001	−20.40 (−23.59 to −17.20)
CRP (mg/L)		0.64 ± 0.89	29.72 ± 13.67	< 0.0001	29.08 (25.25 to 32.92)
ESR (mm/h)		9.66 ± 3.71	30.64 ± 11.48	< 0.0001	20.98 (17.66 to 24.29)
fecal calprotectin (µg/g)		24.30 ± 10.82	363.8 ± 168.10	< 0.0001	339.5 (292.40 to 386.70)
PUCAI		-	30.66 ± 14.05		
Gender				0.492	
male		24 (48%)	46 (54.12%)		
female		26 (52%)	39 (45.88%)		

*P* < 0.05 indicates a significant difference. The data is expressed as mean ± standard deviation, excluding gender

*HC* healthy control, *UC* Ulcerative Colitis, *WBC* white blood cell count, *PLT* platelet count, *Hb* hemoglobin, *CRP* C-reactive protein, *ESR* erythrocyte sedimentation rate, *PUCAI* (Pediatric Ulcerative Colitis Activity Index) is an evaluation of the degree of intestinal inflammation activity in UC patients, including disease-specific indicators such as abdominal pain, rectal bleeding, and bowel frequency; The healthy control group had no symptoms related to UC, so there was no need to test for this indicator

### MiR-340-5p expression is downregulated in UC

**Fig. 1:**
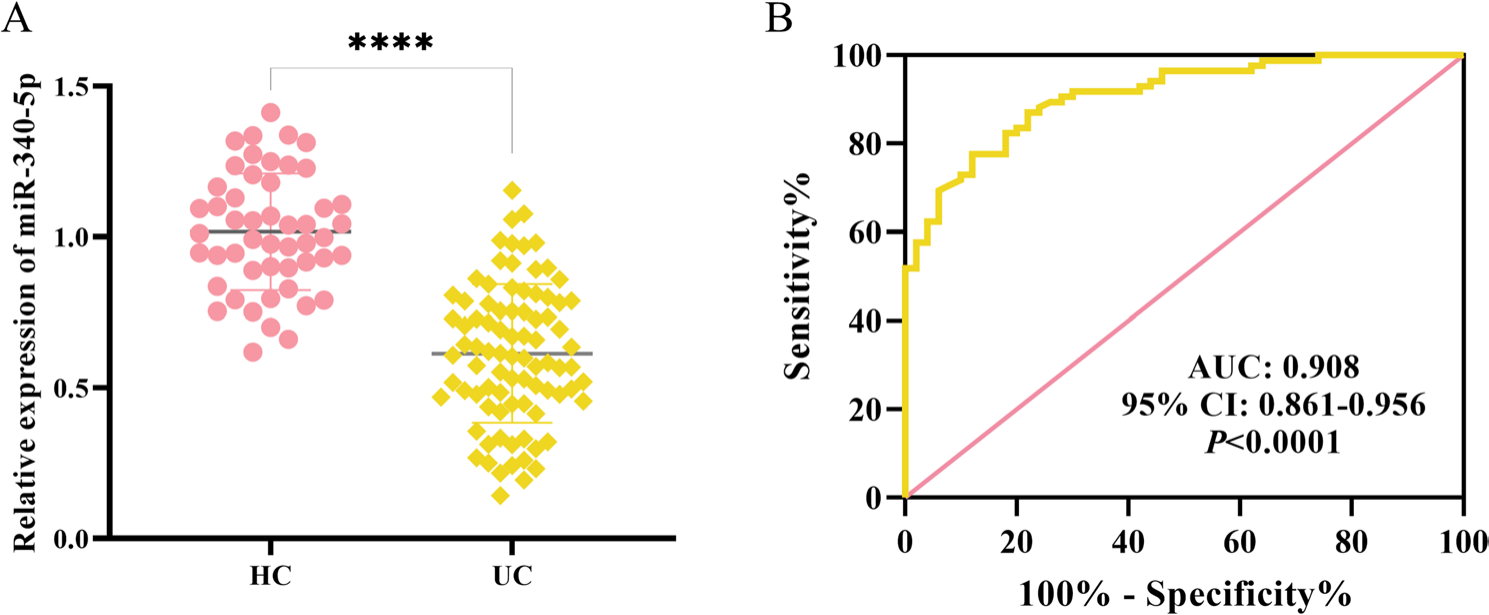
Expression profile of miR-340-5p in the blood of healthy controls and UC patients. **A** Relative expression levels of miR-340-5p. HC: healthy controls; UC: ulcerative colitis, ****P < 0.0001. **B** ROC curve of miR-340-5p

qRT-PCR analysis revealed that miR-340-5p levels were significantly lower in UC patients compared with HC (*P* < 0.0001, Fig. 1A). The AUC of miR-340-5p was 0.908, sensitivity is 77.65%, specificity is 88% and 95% CI is 0.861–0.956, indicating a strong diagnostic potential for UC. (*P* < 0.0001, Fig. 1B).

### Correlation analysis of miR-340-5p

Pearson correlation analysis showed a significant negative correlation between miR-340-5p expression and clinical parameters CRP (*r*=−0.711), ESR (*r*=−0.688), and PUCAI (*r*=−0.691) (*P* < 0.0001; Table 2).

**Table 2:** The correlation between miR-340-5p and clinical features

Factor	Correlation coefficient (*r*)	*P*	95% CI	
			Lower limit	Upper limit
CRP (mg/L)	−0.711	< 0.0001	−0.803	−0.587
ESR (mm/h)	−0.688	< 0.0001	−0.786	−0.556
PUCAI	−0.691	< 0.0001	−0.788	−0.5607

*CRP* C-reactive protein, *ESR* erythrocyte sedimentation rate, *PUCAI* (Pediatric Ulcerative Colitis Activity Index) is an evaluation of the degree of intestinal inflammation activity in UC patients, including disease-specific indicators such as abdominal pain, rectal bleeding, and bowel frequency; The healthy control group had no symptoms related to UC, so there was no need to test for this indicator

### MiR-340-5p targets MAP3K2 regulation

**Fig. 2:**
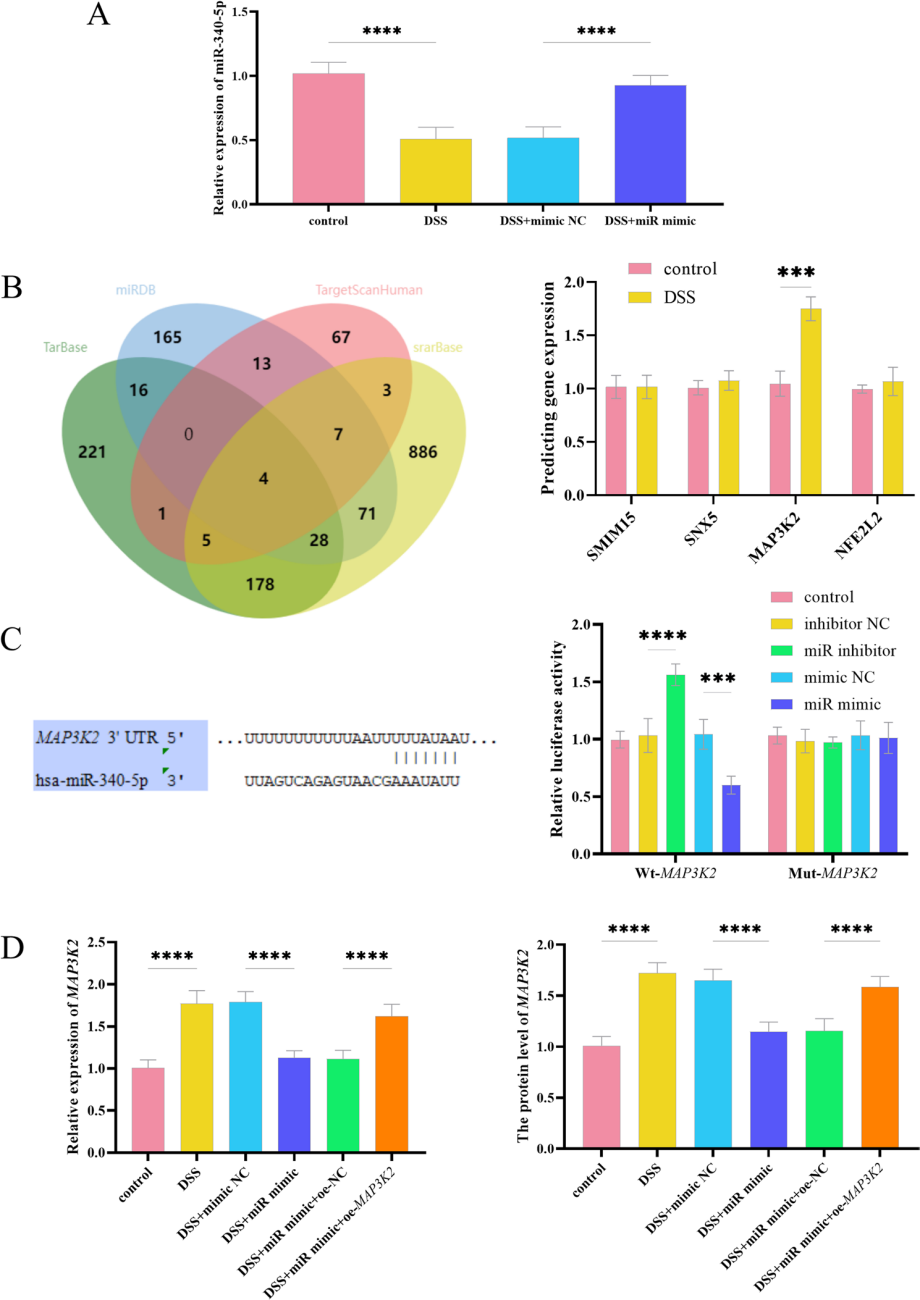
DSS-induced in vitro UC model in HT-29 cells. **A** Expression of miR-340-5p under DSS induction. **B** Venn diagram for predicting downstream target genes of miR and verification of their expression levels. **C** Prediction of binding sites and verification by dual-luciferase assay. **D** miR-340-5p regulates the mRNA and protein expression of *MAP3K2*. NC: negative control; miR: miR-340-5p, ****P < 0.0001, ***P < 0.001, *n* = 3

In vitro experiments in HT-29 revealed that after DSS induction, the relative expression level of miR-340-5p decreased significantly. After adding a miRNA mimic, the expression level was significantly higher than that in the NC control group (*P* < 0.0001, Fig. 2A). Through the prediction and screening of miR-340-5p target genes and the detection of the expression levels of four target genes, it was found that the expression of *MAP3K2* was significantly higher after DSS induction compared with normal treatment (*P* < 0.001, Fig. 2B). Bioinformatics analysis predicted that miR-340-5p directly targets the 3’ untranslated region of *MAP3K2*. As expected, luciferase activity was significantly reduced following transfection with the miR-340-5p mimic into cells containing the WT reporter (*P* < 0.0001). Conversely, the miR-340-5p inhibitor significantly increased luciferase activity (*P* < 0.001). Interestingly, this change disappeared after mutating the binding site (Fig. 2C). DSS induction significantly upregulated *MAP3K2* mRNA and protein expression, whereas miR-340-5p mimic transfection reversed these effects. Ectopic expression of *MAP3K2* attenuated the inhibitory impact of miR-340-5p on *MAP3K2* expression at both transcriptional and translational levels (*P <* 0.0001, Fig. 2D and Figure S1).

### MiR-340-5p reduces DSS-induced cell apoptosis

**Fig. 3:**
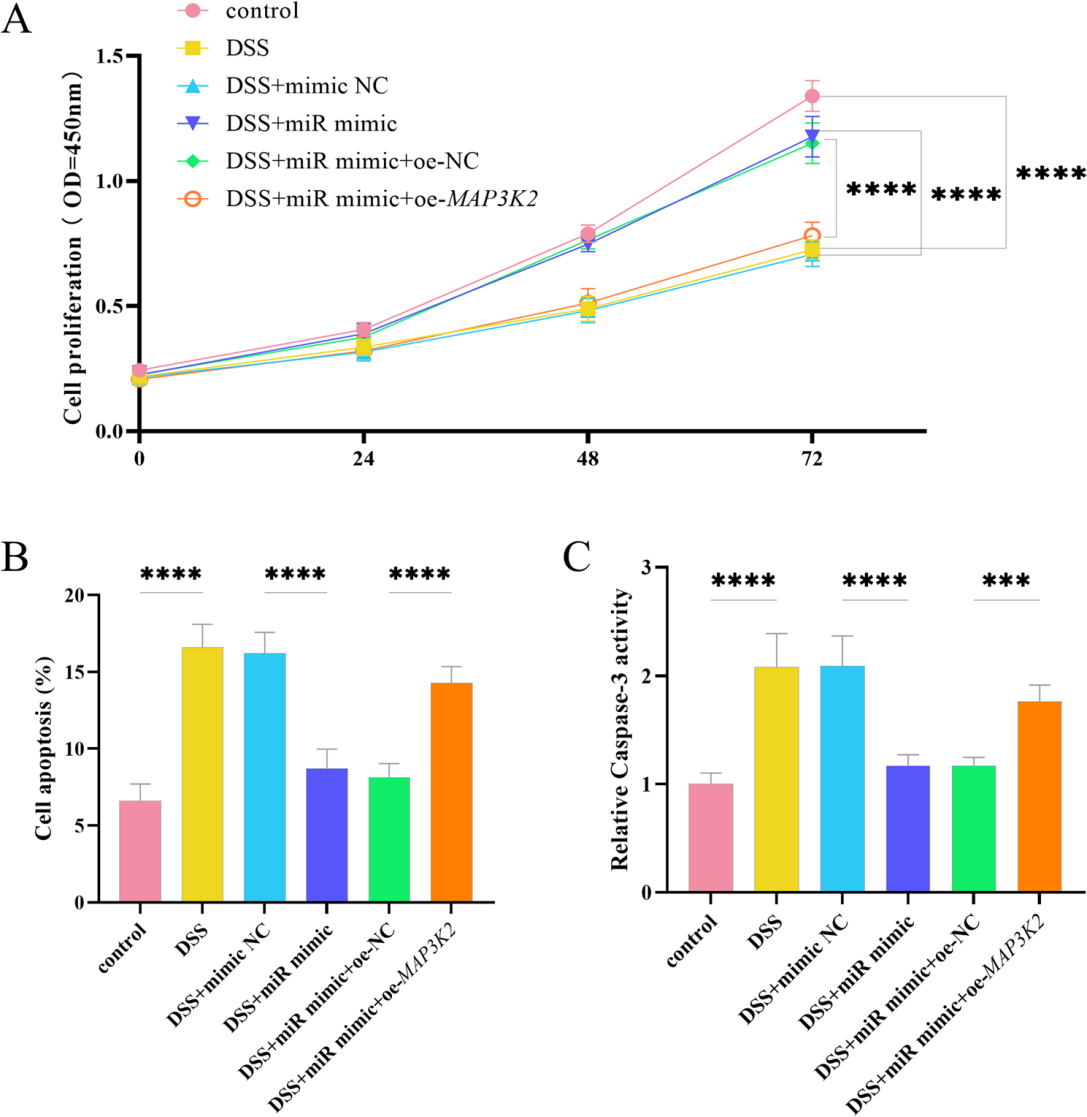
miR-340-5p affects DSS-induced cell viability. **A** Changes in cell proliferation rate. **B** Cell apoptosis rate. **C** Caspase-3 activity assay. NC: negative control; miR: miR-340-5p, ****P < 0.0001, ***P < 0.001, *n* = 3

The cell proliferation rate decreased significantly under DSS induction, and transfection with miR-340-5p mimic significantly increased it (*P* < 0.0001, Fig. 3A). The cell apoptosis rate (Fig. 3B) and the activity of Caspase-3 (Fig. 3C) increased significantly under DSS induction (*P* < 0.0001). Interestingly, adding miR-340-5p alleviated DSS-induced cell apoptosis and the activity of Caspase-3, a key factor in apoptosis (*P* < 0.0001). However, this beneficial effect was counteracted when *MAP3K2* was overexpressed.

### MiR-340-5p affects cellular inflammatory response and oxidative stress through MAP3K2

**Fig. 4:**
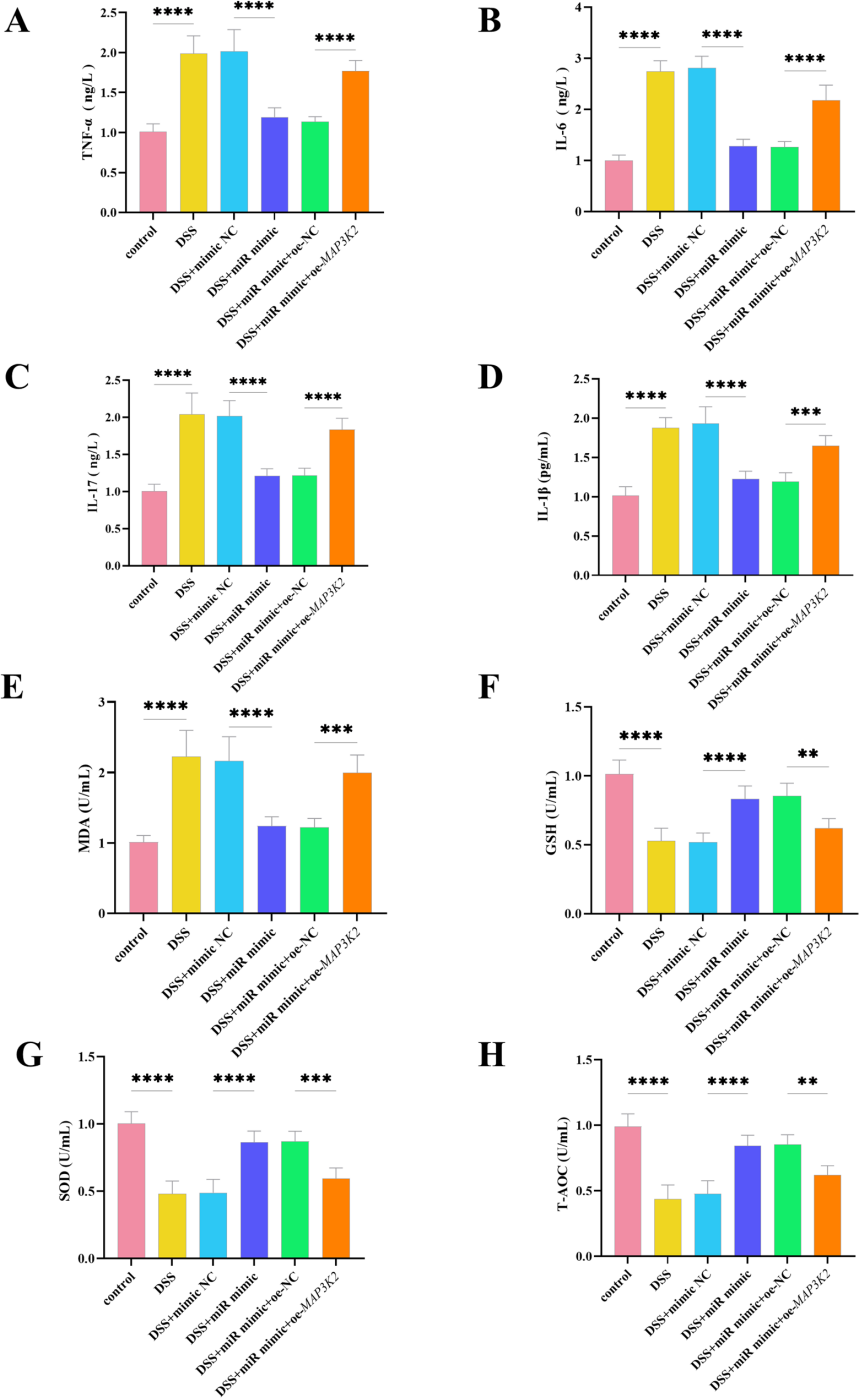
Effects of miR-340-5p on DSS-induced inflammatory response and oxidative stress. **A** Levels of the inflammatory cytokine TNF-α under different treatments. **B** Levels of IL-6 under different treatments. **C** Levels of IL-17 under different treatments. **D** Levels of IL-1β under different treatments. **E** Levels of the oxidative index MDA under different treatments. **F** Levels of the antioxidant GSH under different treatments. **G** Levels of the oxidative index SOD under different treatments. **H** Levels of the antioxidant T-AOC under different treatments. ****P < 0.0001, ***P < 0.001, **P < 0.01, *n* = 3

Following DSS treatment, the concentrations of all pro-inflammatory cytokines were significantly elevated (*P* < 0.0001). The addition of miR-340-5p mimic led to a significant reduction in inflammatory cytokine levels (*P* < 0.001). In cells with *MAP3K2* overexpression, following transfection with miR-340-5p mimic, the concentrations of TNF-α (*P* < 0.001), IL-6 (*P* < 0.0001), IL-17 (*P* < 0.0001), and IL-1β (*P* < 0.001) were significantly elevated compared to the negative control group. (Figs. 4A-D). Under DSS induction, miR-340-5p significantly decreased MDA, an oxidative stress marker, whereas oe-*MAP3K2* led to significantly higher MDA levels compared to the negative control group (Fig. 4E). On the contrary, under DSS induction, GSH, SOD, and T-AOC were significantly reduced. The addition of miR-340-5p mimetics could alleviate this decrease, but oe-*MAP3K2* treatment weakened this relief effect (Fig. 4F-H).

## Discussion

### miR-340-5p is a potential diagnostic biomarker for pediatric UC

Pediatric patients account for approximately 15–20% of all ulcerative colitis cases, highlighting its significant impact on children, making it a critical disease affecting children’s healthy growth and quality of life [35]. In recent years, miRNAs such as miR-654-3p and microRNA-148a-3p have been identified as potential biomarkers for diagnosing inflammatory enteritis [41, 43, 45]. To identify key molecular markers for pediatric UC, this study found that miR-340-5p levels were significantly lower in pediatric UC patients compared with healthy controls. ROC curve analysis further indicated the substantial diagnostic utility of miR-340-5p in pediatric UC. Clinical parameters including the PUCAI, CRP, WBC, and ESR are established indicators for assessing pediatric UC severity and biomarker identification [11, 14, 24]. Our results demonstrated significant inverse correlations between miR-340-5p expression and PUCAI, CRP, as well as ESR. These findings imply that miR-340-5p holds considerable promise as a precise diagnostic biomarker for pediatric UC.

### miR-340-5p participates in pediatric UC by targeting MAP3K2

To explore the molecular mechanisms through which miR-340-5p modulates ulcerative colitis UC, we established DSS-induced HT-29 cell models in vitro to observe its regulatory signaling pathways [4, 6]. In this cellular model, miR-340-5p expression was significantly downregulated following DSS treatment, further indicating its involvement in the pathogenesis of pediatric UC. In recent years, an increasing number of disease studies have utilized gene expression network analysis and screening techniques such as gene mapping [18, 20, 20, 21, 27]. Therefore, combining miRNA gene expression network analysis to screen target genes is more convenient and efficient. TarBase, miRDB, TargetScanHuman, and Starbase Four genes (*SMIMI5*,* SNX5*,* MAP3K2*,* NFE2L2*) were obtained by screening the intersection of four databases. Therefore, they were predicted as targets for miR-340-5p prediction. Tissue specific expression detection revealed differential expression of *MAP3K2*, while the other three genes may not have differential expression due to different regulatory environments or false positive effects of prediction methods. As a key kinase in the MAPK (mitogen-activated protein kinase) signaling pathway, *MAP3K2* has been reported to promote intestinal regeneration by regulating mesenchymal subpopulations in intestinal stromal cells (MRISCs) [40], mediate IL-18-dependent Th1 cell differentiation in the gut [39], and modulate tumor growth in colon cancer [15], suggesting its potential role in UC progression. Bioinformatics analysis predicted a binding site between miR-340-5p and the 3’-UTR of *MAP3K2*, which was experimentally validated via luciferase reporter assays. Subsequent analysis of *MAP3K2* mRNA and protein levels under DSS treatment conditions revealed that miR-340-5p suppressed *MAP3K2* expression in DSS-treated HT-29 cells, confirming that miR-340-5p influences UC pathogenesis by inhibiting *MAP3K2*. Although the interaction between miR-340-5p and *MAP3K2* has been previously documented in other pathological contexts, such as endometriosis [36] and acute exacerbation of COPD [47], its role in ulcerative colitis, particularly in the pediatric population, remains entirely unexplored. To our knowledge, this study is the first to identify and functionally characterize the miR-340-5p/*MAP3K2* axis in pediatric UC. Based on previous research, we speculate that under the inflammatory conditions of UC, *MAP3K2*-mediated IL-18-dependent Th1 cell differentiation significantly promotes local intestinal inflammatory responses, while miR-340-5p, through its targeted inhibition of *MAP3K2*, effectively blocks this immune differentiation pathway, thereby alleviating colonic inflammation. Simultaneously, as an upstream regulator in the MAPK signaling pathway, the suppression of *MAP3K2* expression also influences downstream signaling transduction related to apoptosis and oxidative stress. These findings not only reveal the pivotal role of this axis in coordinating inflammation, apoptosis, and oxidative stress in pediatric UC but also highlight its potential translational value as a therapeutic target for this condition.

### Molecular mechanisms of miR-340-5p/MAP3K2 in regulating cell apoptosis, inflammation, and oxidative stress

Cell functional assays demonstrated that miR-340-5p can reverse DSS-induced exacerbated cell apoptosis and proliferation inhibition in HT-29 cells while reducing inflammatory responses and oxidative stress damage under adverse conditions. This finding aligns with previous research on miR-340-5p’s protective effects against adverse reactions in diseases such as myocarditis and diabetic retinopathy [22, 38, 44]. The downstream target gene *MAP3K2* is known to exert essential functions in both physiological and pathological processes, including cell apoptosis, inflammatory responses, and oxidative stress [9]. Importantly, the protective effects of miR-340-5p mimic were largely abolished by co-overexpression of *MAP3K2*, as evidenced by aggravated apoptosis, increased pro-inflammatory cytokines, and enhanced oxidative damage compared to the group transfected with miR-340-5p mimic alone. Inflammatory factors TNF -α, IL-1β, IL-6 are associated with the UC activator NF-kB pathway, leading to diarrhea, abdominal pain, bleeding, and many extraintestinal manifestations [12]. The inflammation of IL-17 forms a unique fibroblast population in UC disease [32]. miR-340-5p regulates cell apoptosis, affects the secretion of inflammatory factors such as TNF-α, IL-6, IL-17, and IL-1β, as well as oxidative stress levels such as MDA, GSH, SOD, and T-AOC by targeting *MAP3K2*, thereby affecting the disease progression of pediatric UC. Our results indicate the first identification of the “miR-340-5p-*MAP3K2*” regulatory axis in pediatric UC, uncovering its diverse roles in inflammation, oxidative stress, and apoptotic processes. These findings lay a foundation for further exploring the in vivo regulatory pathways of miR-340-5p/*MAP3K2*. Targeted intervention of miR-340-5p may circumvent the systemic side effects of traditional immunosuppressants, offering a novel strategy for developing precision therapies for pediatric UC.

### Limitations and future prospects

Although this study elucidated the regulatory mechanism of miR-340-5p in an in vitro model, there are certain limitations. (1) This study lacks in vivo validation, and in the future, a UC mouse model will be constructed to further validate the regulatory molecular mechanisms, strengthen clinical dynamic detection, and analyze the changes in miR-340-5p expression before and after treatment for clinical diagnosis and patient care. (2) The functional research of *MAP3K2* has not been completed yet. In the future, further exploration will be conducted on downstream regulatory factors to elucidate the specific molecular mechanisms of the miR-340-5p/*MAP3K2* pathway. (3) Due to regional differences in genetic background, environmental exposure, dietary habits, and clinical diagnosis and management practices, the performance of diagnostic biomarkers may vary among different populations. In future research, diverse populations will be included, cross regional and multi center studies will be conducted, and correction factors/algorithms will be developed and validated.

## Conclusions

This study demonstrated that miR-340-5p exhibits strong diagnostic potential in pediatric UC, with its expression showing a significant negative correlation with disease activity. Mechanistically, miR-340-5p directly targets *MAP3K2*, thereby reducing inflammatory responses, cell apoptosis, and oxidative stress damage. These findings offer new perspectives on the pathogenesis of pediatric UC, underscoring the essential role of the miR-340-5p/*MAP3K2* regulatory axis in disease development.

## Supplementary Information

Supplementary Material 1.

Supplementary Material 2.

## Data Availability

The datasets used and/or analysed during the current study are available from the corresponding author on reasonable request.

## Abbreviations

ASUC: Acute severe ulcerative colitis.
CRP: C-reactive protein
ESR: Erythrocyte sedimentation rate
miRNAs: MicroRNAs
PUCAI: Pediatric Ulcerative Colitis Activity Index
UC: Ulcerative colitis
