# Correction: The diagnostic value of miR-340-5p in pediatric ulcerative colitis and its molecular mechanism by targeting *MAP3K2* to modulate intestinal epithelial cell dysfunction

**DOI:** 10.1186/s41065-026-00652-3

**Published:** 2026-02-24

**Authors:** Fanting Meng, Xianlong Han, Lingxia Ge, Nan Guan

**Affiliations:** 1 Department of Gastroenterology, Children’s Hospital of Nanjing Medical University, Nanjing City, Jiangsu Province 210000 China; 2 Department of Anorectal, Qingdao Hiser Hospital Affiliated of Qingdao University (Qingdao Traditional Chinese Medicine Hospital), Qingdao City, Shandong Province 266033 China; 3 Department of Obstetrics and Gynecology, Guangdong Provincial Key Laboratory of Major Obstetric Diseases, Guangdong Provincial Clinical Research Center for Obstetrics and Gynecology, Guangdong-Hong Kong-Macao Greater Bay Area Higher Education Joint Laboratory of Maternal-Fetal Medicine, Guangzhou City, Guangdong Province China; 4 Department of Pediatrics, Guangzhou Key Laboratory of Neonatal Intestinal Diseases, The Third Affiliated Hospital of Guangzhou Medical University, Guangzhou City, Guangdong Province China; 5 Department of Pediatrics, Affiliated Hospital of Gansu Medical College, No.296, Kongtong East Road, Pingliang City, Gansu Province China

**Correction: Hereditas 162, 229 (2025)**

**https://doi.org/10.1186/s41065-025-00597-z**

Following publication of the original article [1], the author reported that Figure 4 image and its caption is not the final version. Additionally, the header of Supplementary Table 1 needs to be updated from Table S2 to Table S1.

**Incorrect Figure 4**


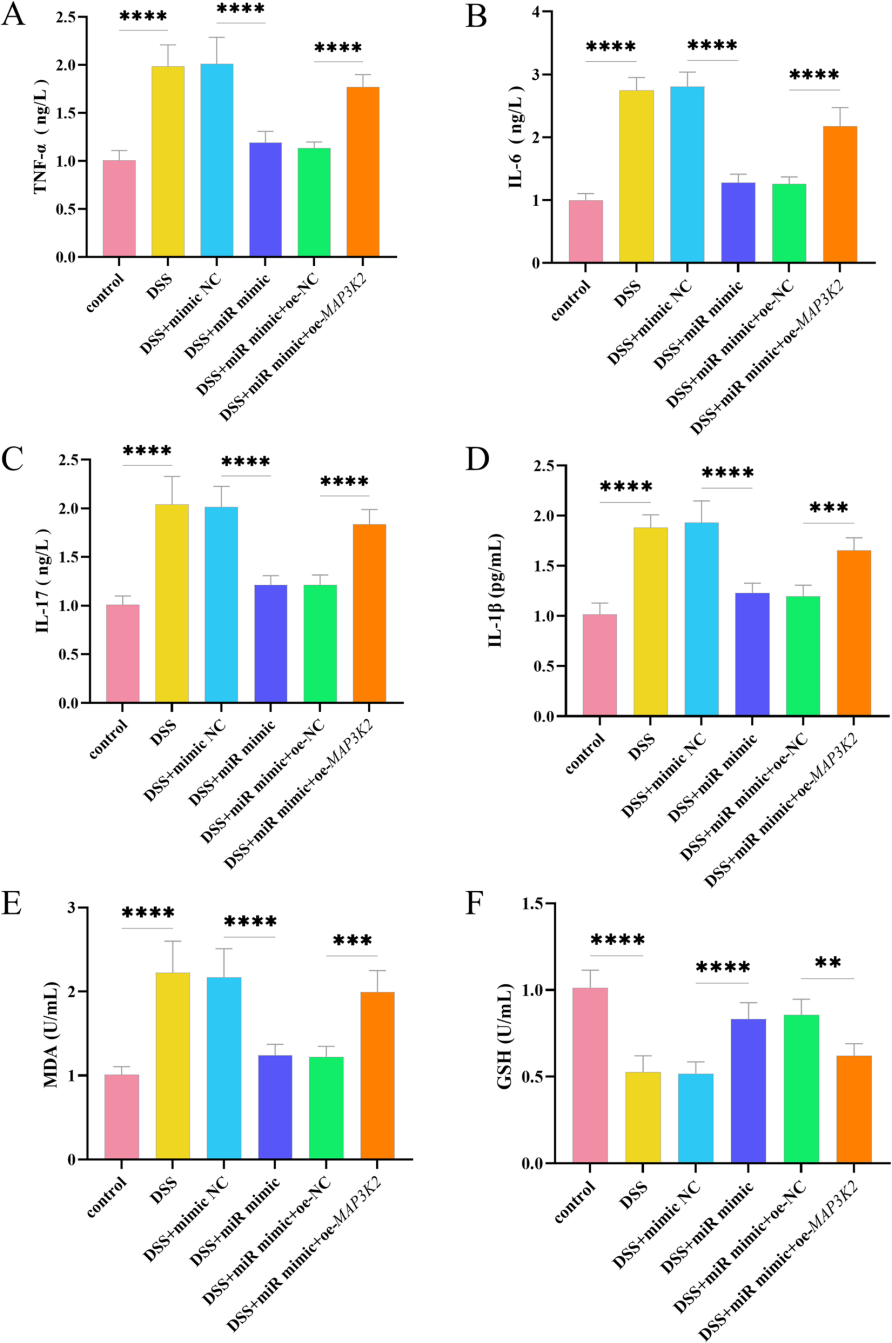


**Correct Figure 4**

**Fig. 4:**
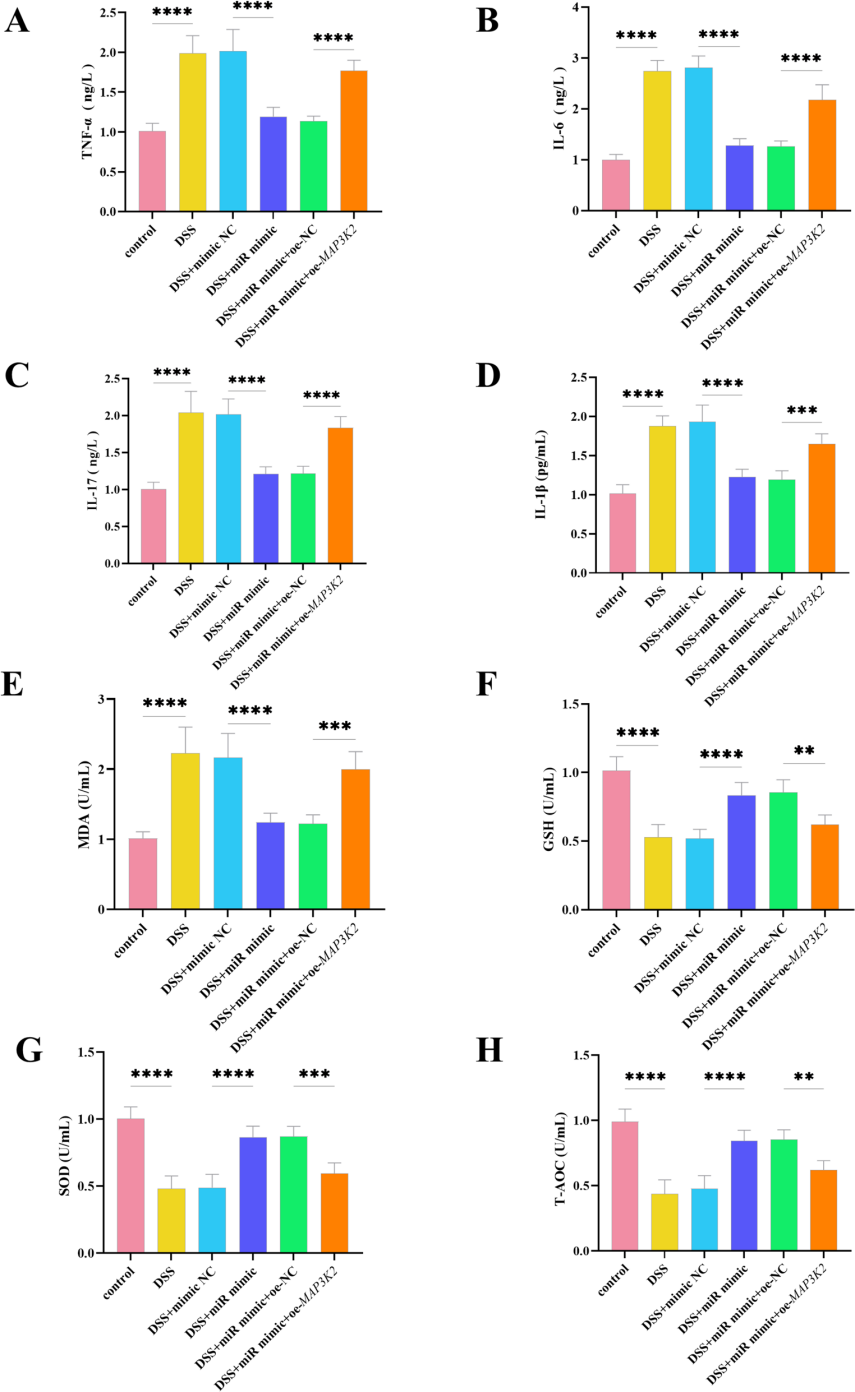
Effects of miR-340-5p on DSS-induced inflammatory response and oxidative stress. **A** Levels of the inflammatory cytokine TNF-α under different treatments. **B** Levels of IL-6 under different treatments. **C** Levels of IL-17 under different treatments. **D** Levels of IL-1β under different treatments. **E** Levels of the oxidative index MDA under different treatments. **F** Levels of the antioxidant GSH under different treatments. **G** Levels of the oxidative index SOD under different treatments. **H** Levels of the antioxidant T-AOC under different treatments. ****P <0.0001, ***P <0.001, **P <0.01, *n*=3. ****P <0.0001, ***P <0.001, **P <0.01, *n*=3

The original article has been updated.
