# Supplementary material for: The diagnostic value of miR-340-5p in pediatric ulcerative colitis and its molecular mechanism by targeting MAP3K2 to modulate intestinal epithelial cell dysfunction
Source: Hereditas. 2025 Nov 19;162:229. doi: 10.1186/s41065-025-00597-z (PMC12628879; doi:10.1186/s41065-025-00597-z)
Supplement: Supplementary file 1 — Supplementary Material 1. [file 41065_2025_597_MOESM1_ESM.docx]

**Table S1.** Primer sequences used in RT-qPCR

| **Gene** | **Sequence** |
| --- | --- |
|  |  |
| **miR-340-5p forward** | CACTCCAGCTGGGTTATAAAGCAATGAGA |
| **miR-340-5p reverse** | TGGTGTCGTGGAGTCG |
| ***MAP3K2* forward** | GCTTACGGTCTCCTGTGAGTT |
| ***MAP3K2* reverse** | AGGATTGTCTATGTCACTTCCCC |
| ***GAPDH* forward** | GAGTCAACGGATTTGGTCGT |
| ***GAPDH* reverse** | TTGATTTTGGAGGGATCTCG |
| **U6 forward** | CTCGCTTCGGCAGCACA |
| **U6 reverse** | AACGCTTCACGAATTTGCGT |
