# Supplementary figures and images for: The diagnostic value of miR-340-5p in pediatric ulcerative colitis and its molecular mechanism by targeting MAP3K2 to modulate intestinal epithelial cell dysfunction
Source: Hereditas. 2025 Nov 19;162:229. doi: 10.1186/s41065-025-00597-z (PMC12628879; doi:10.1186/s41065-025-00597-z)

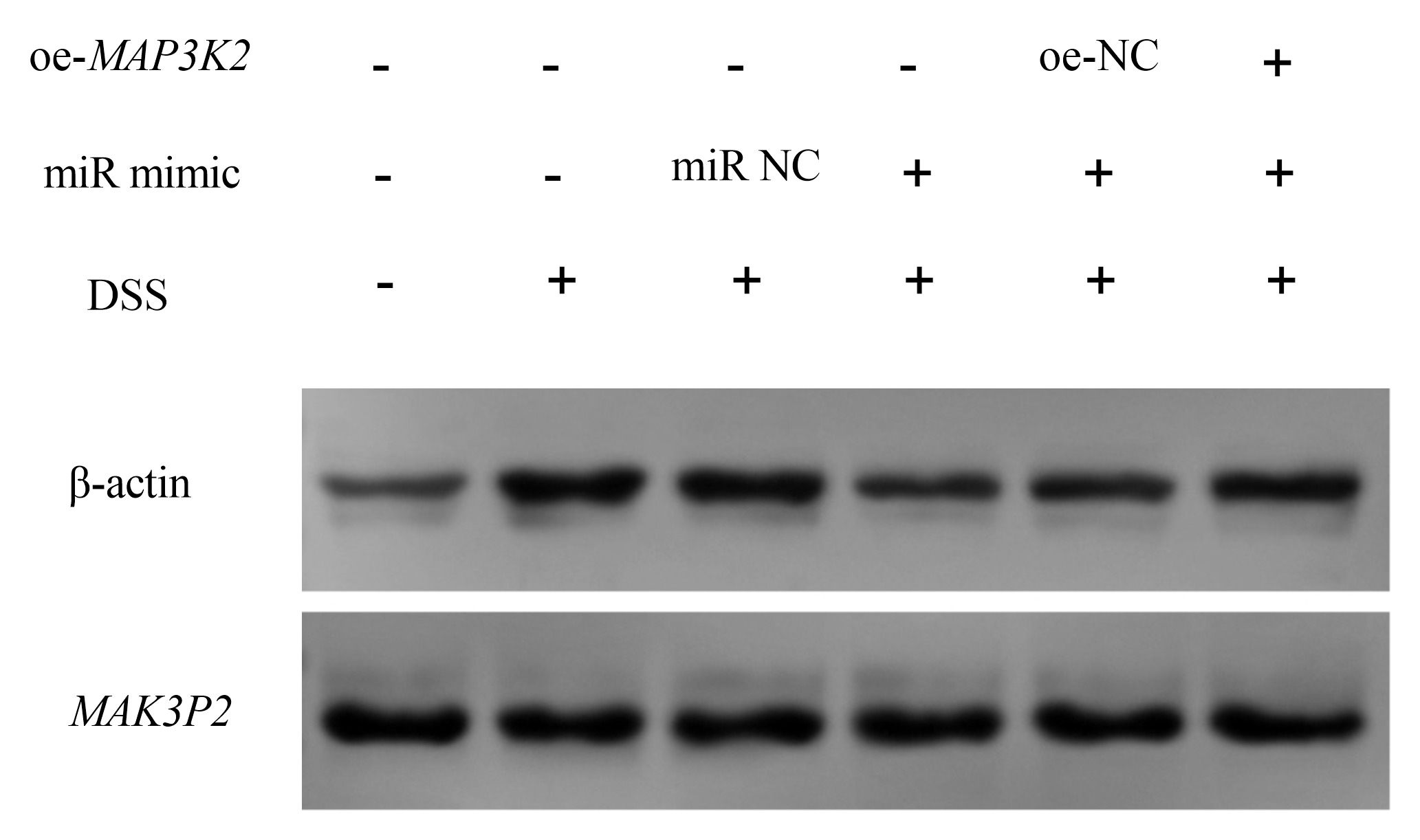

Supplement: Supplementary file 2 — Supplementary Material 2. [file 41065_2025_597_MOESM2_ESM.tif]
